# Inflammatory myoglandular polyp of the cecum: case report and review of literature

**DOI:** 10.1186/1471-230X-10-10

**Published:** 2010-01-26

**Authors:** Roberto L Meniconi, Roberto Caronna, Michele Benedetti, Gianfranco Fanello, Antonio Ciardi, Monica Schiratti, Federica Papini, Francesco Farelli, Giuseppe Dinatale, Piero Chirletti

**Affiliations:** 1 Department of Surgery "F. Durante" General Surgery N, Sapienza University of Rome, Viale del Policlinico 155, 00161, Rome, Italy; 2 Department of Surgery "P. Valdoni", Surgical Pathology Unit, Sapienza University of Rome, Viale del Policlinico 155, 00161, Rome, Italy; 3 General Surgery Residency V, Sapienza University of Rome, Italy

## Abstract

**Background:**

Inflammatory myoglandular polyp (IMGP) is a rare non-neoplastic polyp of the large bowel, commonly with a distal localization (rectosigmoid), obscure in its pathogenesis. Up till now, 60 cases of IMGP have been described in the literature, but none located in the cecum.

**Case presentation:**

We report a case of a 53-year-old man who was admitted to our hospital for further evaluation of positive fecal occult blood test associated to anemia. A colonoscopy identified a red, sessile, lobulated polyp of the cecum, 4.2 cm in diameter, partially ulcerated. The histological examination of the biopsy revealed the presence of inflammatory granulation tissue with lymphocytic and eosinophil infiltration associated to a fibrous stroma: it was diagnosed as inflammatory fibroid polyp. Considering the polyp's features (absence of a peduncle and size) that could increase the risk of a polypectomy, a surgical resection was performed. Histological examination of the specimen revealed inflammatory granulation tissue in the lamina propria, hyperplastic glands with cystic dilatations, proliferation of smooth muscle and multiple erosions on the polyp surface: this polyp was finally diagnosed as IMGP. There was also another little polyp next to the ileocecal valve, not revealed at the colonoscopy, 0.8 cm in diameter, diagnosed as tubulovillous adenoma with low grade dysplasia.

**Conclusions:**

This is the first case of IMGP of the cecum. It is a benign lesion of unknown pathogenesis and must be considered different from other non-neoplastic polyps of the large bowel such as inflammatory cap polyps (ICP), inflammatory cloacogenic polyps, juvenile polyps (JP), inflammatory fibroid polyps (IFP), polyps secondary to mucosal prolapse syndrome (MPS), polypoid prolapsing mucosal folds of diverticular disease. When symptomatic, IMGP should be removed endoscopically, whereas surgical resection is reserved only in selected patients as in our case.

## Background

Inflammatory myoglandular polyp (IMGP) is a rare non-neoplastic polyp of the large bowel, commonly with a distal localization but it has been also described in the descending and transverse colon [1-11]. IMGP can be asymptomatic [6] or show non-specific symptoms such as positive fecal occult blood, hematochezia or chronic anemia [1-13]. Its pathogenesis is still unclear but we believe that its histological patterns make it a distinct entity [14]. We described the first case of IMGP of the cecum and also we made a review of the literature in order to evaluate clinical, diagnostic and therapeutic issues.

## Case presentation

**Figure 1:**
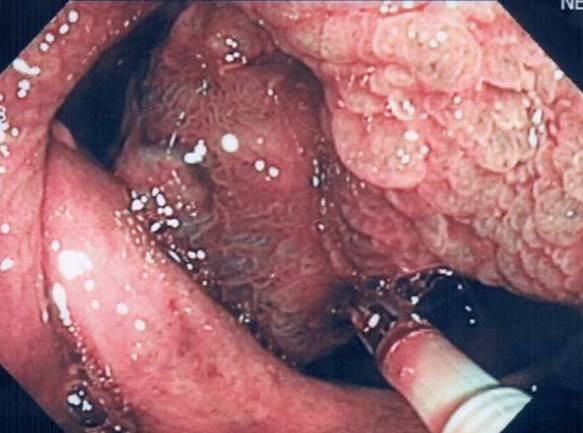
**Endoscopic view**. Colonoscopy showing a red, sessile and lobulated polyp in the cecum. A biopsy is performed.

**Figure 2:**
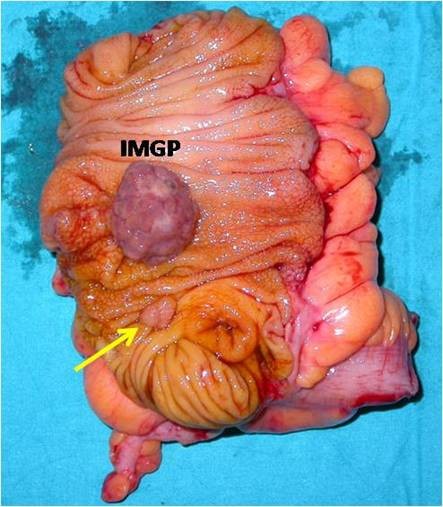
**Surgical specimen: ileocecal resection**. Surgical specimen: ileocecal resection. IMGP appears to be a spherical, sessile and lobulated polyp of the cecum, without a peduncle, 4.2 cm in diameter. An arrow shows a little adenomatous polyp below the IMGP next to the ileocecal valve.

**Figure 3:**
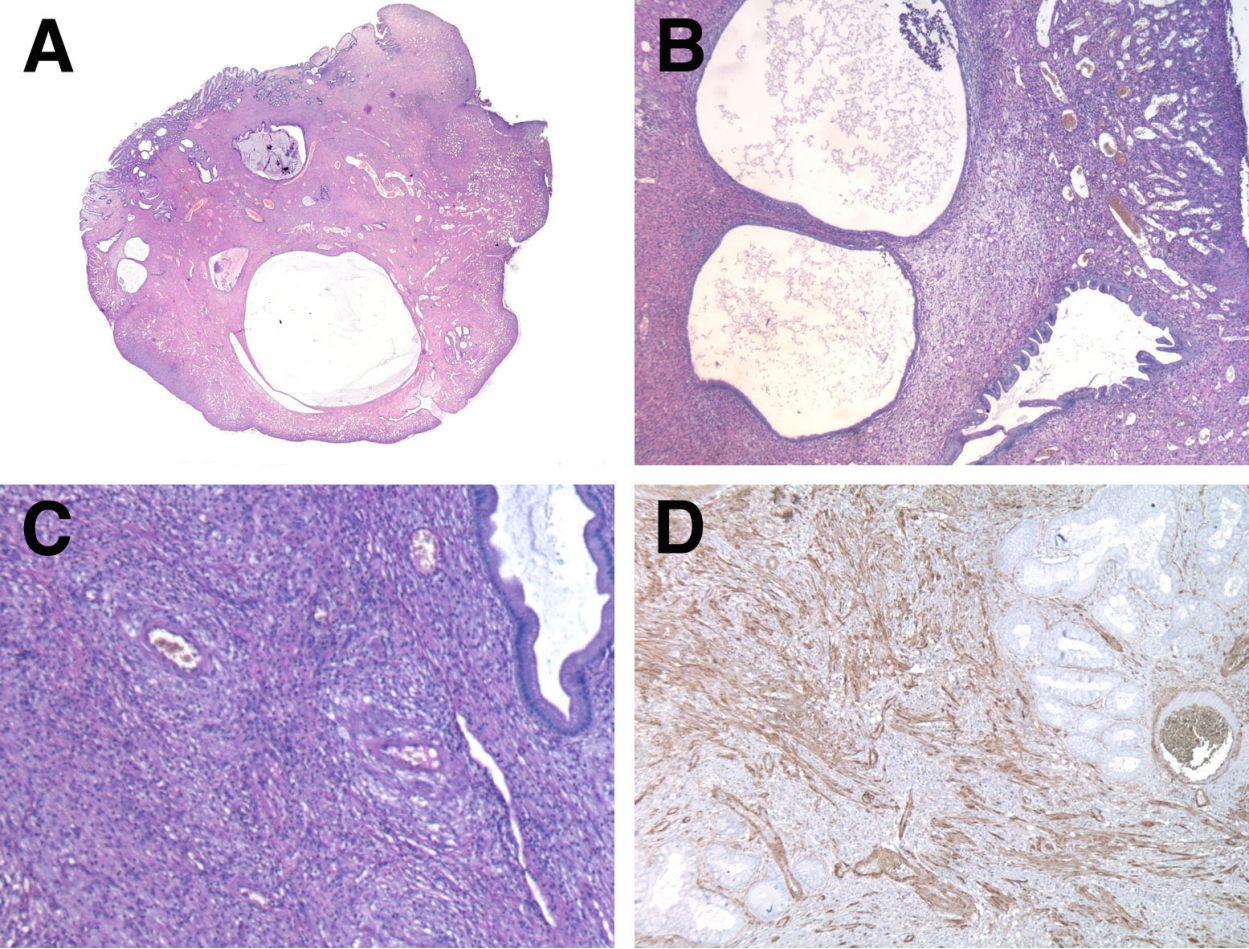
**Histological findings**. Polyp's whole picture, showing cystic dilatations embedded in the fibromuscular stroma (A). Low power view of inflammatory granulation tissue and engorged capillaries in the lamina propria, hyperplastic glands with cystic dilatations and proliferation of smooth muscle from muscolaris mucosae (B, C). Diffuse positive immunostaining of smooth muscle actin (D). (A, B, C: hematossilin-eosin staining, A 1:1, B 2,5× obj, C 4× obj; D: anti-actin immunoperoxidase, 4× obj).

A 53-year-old man was admitted to our hospital for further evaluation of anemia (haemoglobin concentration 10,6 g/dl, RBC 2.970.000/mm^3^, HTC 31,4%) associated to positive fecal occult blood test. He had been treated for an autoimmune haemolytic anemia 4 years before, but immuno-haematological tests excluded signs of haemolysis. He also had an autoimmune thyroiditis. He had neither bleeding from rectum nor diarrhea or other gastrointestinal symptoms. Abdominal examination was negative. Rectal exploration did not reveal any pathological sign. A colonoscopy identified the presence of a spherical, sessile, lobulated polyp of the cecum, 4.2 cm in diameter, with a red and not bleeding but partially ulcerated surface (figure 1). Because of the absence of a peduncle and its large size, the endoscopist decided to perform a biopsy of the polyp rather than a polypectomy. The histological examination of the specimen revealed the presence of inflammatory granulation tissue with lymphocytic and eosinophil infiltration associated to a fibrous stroma: it was diagnosed as inflammatory fibroid polyp [15]. Considering the chronic anemia and the polyp's features (absence of a peduncle and size), we decided to perform an ileocecal resection with a latero-lateral ileocolic anastomosis (figure 2). The histological examination of the polyp revealed inflammatory granulation tissue and engorged capillaries in the lamina propria, hyperplastic glands with cystic dilatations, proliferation of smooth muscle and multiple erosions on the polyp surface (figure 3). There was not superficial fibrin exudation. According to these features [1] it was finally diagnosed as inflammatory myoglandular polyp (IMGP). There was also another little polyp next to the ileocecal valve, not revealed at the colonoscopy, 0.8 cm in diameter, diagnosed as tubulovillous adenoma with low grade dysplasia. The patient was discharged from our hospital four days after the operation without any postoperative complications. After two months the patient has good health and anemia was resolved.

## Discussion

Nakamura and colleagues first described IMGP in 1992 as a non-neoplastic colorectal polyp with three main histological features: 1) inflammatory granulation tissue in the lamina propria; 2) proliferation of smooth muscle from the muscolaris mucosae; 3) hyperplastic glands with occasional cystic dilatation [1]. A review of literature on MEDLINE revealed only 60 cases of IMGPs reported from 1992 to 2009 [1-13].

IMGP can be asymptomatic [1,4,6,8,11], whereas hemorrage is the most common symptom: it can develop as positive fecal occult blood [1,5,7,8,11], hematochezia [1,5,7-10,12,13] or anemia [1,8]. Other non-specific symptoms may be abdominal pain or constipation [1,8]. In our case the patient was investigated for positive fecal occult blood, although he had signs of chronic anemia for several months before the admission to our hospital.

Large bowel is the only gastrointestinal tract where IMGP has been described, with the exception of the case in the terminal ileum reported by Griffiths et al. [2]. It is mostly located in the left colon: of 60 cases, 9 (15%) were in the rectum, 30 (50%) in the sigma, 6 (10%) in the descending colon, 13 (21,6%) in the transverse colon, 1 (1,7%) in the ascending colon, 1 (1,7%) in the ileum [1-13]. We reported the first case of IMGP of the cecum. According to the literature the endoscopy can show a pedunculated (88,3%) or sessile polyp (11,7%), with a smooth (90%) or lobulated (10%) surface [1-13]. The polyp we found appears to be rare because it is a sessile polyp with a lobulated surface and superficial erosions.

The final diagnosis of IMGP is achieved by histological examination through endoscopic mucosal resection (EMR) or polypectomy, whereas the simple biopsy alone is not enough for a correct diagnosis as in our case. Some authors [8,12] reported a correlation between the characteristic surface of the polyp under magnifying endoscopy and its histological features. As their experience is limited to 10 patients more cases should be studied.

IMGP must be differentiated from other non-neoplastic polyps such as inflammatory cap polyps (ICP), inflammatory cloacogenic polyps, juvenile polyps (JP), inflammatory fibroid polyps (IFP), polyps secondary to mucosal prolapse syndrome (MPS), polypoid prolapsing mucosal folds of diverticular disease [6]. Bathal et al. [14,16] consider IMGP and all these lesions as part of a same group with small histological variations and as result of prolapse. However, we agree with others [1,9-18] who consider IMGP as a distinct entity. In fact, each type of these polyps presents both histological and clinical differences from the IMGP. Inflammatory cap polyps (ICP) are usually multiple, sessile, covered by a fibrin cap and associated with inflammatory bowel diseases or colonic cancer [19]. Moreover the common symptoms of ICPs are mucous diarrhea and tenesmus, and they usually develop in the rectosigma. Inflammatory cloacogenic polyps are solitary, peduncolated, with tubulovillous architecture and localized only at the anorectal transition zone [20]. Juvenile polyps do not show proliferation of the muscolaris mucosae and develop in young age [1]. Inflammatory fibroid polyps (IFP) mostly occur in stomach and small bowel and are histologically characterized by connective tissue with abundant inflammatory cells, in particular plasma cells and eosinophils [15]. IMGP should not be also considered as polyps secondary to mucosal prolapse syndrome or as polypoid prolapsing mucosal folds of diverticular disease. In the former, polypoid lesions are usually villous, granular or cauliflower-like, frequently found in females and in older patients than IMGPs [1]. In the latter, no correlation between IMGP and diverticular disease has been found [21].

Concerning therapy, endoscopic treatment of IMGP is the gold standard because it is clinically and histologically benign as reported in literature [1-9,11-13], with the exception of Kayhan et al. [10] who performed a left hemicolectomy because of the polyp size (> 6 cm). We also decided for surgical resection because of increased risk of endoscopic polypectomy (bleeding and perforation) related to polyp diameter and the absence of a peduncle. However we agree with authors who consider that the number of surgical resections for colonic IMGP will decrease and endoscopic resection will increase in the future considering the recent advances of the diagnostic and therapeutic endoscopy [12,13].

The causes of IMGP are still unclear. Nakamura et al. [1] think that chronic trauma from the fecal stream and from peristalsis may have a role in its pathogenesis. This theory came from the observation that IMGP were found only in the left colon where the feces are solid and more able to injure the colonic mucosa [1,8]. Nevertheless, recent papers described IMGPs in the proximal transverse [7,11] and ascending colon [12] or in the cecum as we found.

## Conclusion

We reported the first case of sessile and lobulated IMGP of the cecum. Concerning its benign biology, it should be removed endoscopically when it is symptomatic (hemorrage, occlusion). Surgical treatment is reserved only in selected cases. It must be considered different from other non-neoplastic polyps for its clinical and histopathological features. More studies are required to understand its pathogenesis.

## Consent

Written informed consent was obtained from the patient for publication of this case report and any accompanying images. A copy of the written consent is available for review by the Editor-in-Chief of this journal

## Competing interests

The authors declare that they have no competing interests.

## Authors' contributions

RLM collected clinical data, reviewed the literature and drafted the manuscript; RC and MB reviewed the manuscript for intellectual content; MS, FP and FF were involved in the care of the patient; AC studied the specimen; GF performed the endoscopy; PC supervised the whole process. All authors read and approved the final manuscript.

## Pre-publication history

The pre-publication history for this paper can be accessed here:

http://www.biomedcentral.com/1471-230X/10/10/prepub
